# Perceptions and experiences with district health information system software to collect and utilize health data in Bangladesh: a qualitative exploratory study

**DOI:** 10.1186/s12913-020-05322-2

**Published:** 2020-05-26

**Authors:** Tahmina Begum, Shaan Muberra Khan, Bridgit Adamou, Jannatul Ferdous, Muhammad Masud Parvez, Mohammad Saiful Islam, Feroza Akhter Kumkum, Aminur Rahman, Iqbal Anwar

**Affiliations:** 1 Health system and population studies division (HSPSD), icddr,b, Dhaka, Bangladesh; 2 Carolina Population Center, University of North Carolina, Chapel Hill, USA; 3 UNICEF, Dhaka, Bangladesh

**Keywords:** Electronic health management information system, District health information system software 2, Facilitators, Barriers, Bangladesh

## Abstract

**Background:**

Accurate and high-quality data are important for improving program effectiveness and informing policy.

In 2009 Bangladesh’s health management information system (HMIS) adopted the District Health Information Software, Version 2 (DHIS2) to capture real-time health service utilization data. However, routinely collected data are being underused because of poor data quality and reporting. W**e** aimed to understand the facilitators and barriers to implementing DHIS2 as a way to retrieve meaningful and accurate data for reproductive, maternal, newborn, child, and adolescent health (RMNCAH) services.

**Methods:**

This qualitative study was conducted in two districts of Bangladesh from September 2017 to 2018. Data collection included key informant interviews (*n* = 11), in-depth interviews (*n* = 23), and focus group discussions (*n* = 2). The study participants were involved with DHIS2 implementation from the community level to the national level. The data were analyzed thematically.

**Results:**

DHIS2 could improve the timeliness and completeness of data reporting over time. The reported facilitating factors were strong government commitment, extensive donor support, and positive attitudes toward technology among staff. Quality checks and feedback loops at multiple levels of data gathering points are helpful for minimizing data errors. Introducing a dashboard makes DHIS2 compatible to use as a monitoring tool. Barriers to effective DHIS2 implementation were lack of human resources, slow Internet connectivity, frequent changes to DHIS2 versions, and maintaining both manual and electronic system side-by-side. The data in DHIS2 remains incomplete because it does not capture data from private health facilities. Having two parallel HMIS reporting the same RMNCAH indicators threatens data quality and increases the reporting workload.

**Conclusion:**

The overall insights from this study are expected to contribute to the development of effective strategies for successful DHIS2 implementation and building a responsive HMIS. Focused strategic direction is needed to sustain the achievements of digital data culture. Periodic refresher trainings, incentives for increased performance, and an automated single reporting system for multiple stakeholders could make the system more user-friendly. A national electronic health strategy and implementation framework can facilitate creating a culture of DHIS2 use for planning, setting priorities, and decision making among stakeholder groups.

## Background

A management information system (MIS), one of the six building blocks of a health system, is essential for strategic planning, priority setting, and decision making [1]. In contrast to a paper-based system, electronic-health (e-health) provides timely and accurate collection of health data leading to better health care planning and improved diagnosis [2]. Electronic health records are often classified into two main categories: individual (i.e., client) records and the records used for information management and decision making. The District Health Information System (DHIS) falls under the latter category of e-health which was first introduced by the University of Oslo in 1994 [3]. A significant initiative under the umbrella of DHIS was the introduction of DHIS Version 2 (DHIS2) software [4]. DHIS2 is an integrated, open-source and web-based platform for health data collection, validation, analysis, and presentation of aggregated and individual data [5, 6]. It aims to improve health service delivery by strengthening the health management information system (HMIS) [7].

Currently, DHIS2 has been translated into multiple languages and 46 countries are using this platform for their HMIS [3]. The system is particularly helpful for a developing country’s health sector to facilitate using their limited resources for evidence-based decision making [8]. Easy aggregation of reproductive, maternal, newborn, child, and adolescent health (RMNCAH) data using DHIS2 has proven to be a supportive factor for effective strategic planning, priority setting, and decision making in many developing countries [9, 10]. Evidence from Uganda and Kenya shows that implementation of DHIS2 has improved reporting of immunization coverage, antenatal care (ANC) visits, and facility delivery rate [11, 12]. In Laos, the effective implementation of DHIS2 on maternal and child health (MNCH) surveillance data improved service delivery through identification of service coverage, barriers to access to services, and causes of maternal death [13]. In Sri Lanka, using DHIS2 data for MNCH information management has also improved quality of care [5].

Bangladesh’s HMIS is considered an active contributor in the global DHIS2 implementation strategy [14]. Currently, about 75% of public health facilities are covered under the DHIS2 network [15]. DHIS2 collects aggregated data on logistic supplies, procurement, human resources, and health indicators, with particular attention placed on MNCH data. A comprehensive list of RMNCAH indicators are collected in the system to track progress. (The common RMNCAH indicators retrieved through DHIS2 are listed under Additional file 1). RMNCAH services in Bangladesh are offered by two directorates under the Ministry of Health and Family Welfare (MOHFW): the Directorate General of Health Services (DGHS) and the Directorate General of Family Planning (DGFP) [14]. The two directorates use different HMIS and only DGHS uses DHIS2. However, the vast majority of private facilities do not report to the national HMIS [15] because the private health system in Bangladesh is not obligated to do so. The data flow system under the DGHS and DGFP is presented as Additional files 2 and 3 respectively. As of February 2019, the data reporting rate through DHIS2 was 98%. Though the reporting rate is deemed satisfactory, the quality of the DHIS2 data in general is considered poor and incomplete [16]. In some instances data from peripheral-level health facilities takes an average of 3 months to reach the central office [16]. The delay in reporting and poor data quality issues force policy makers and health programmers to rely on periodic surveys instead of DHIS2 data [16]. To elaborate further, a UNICEF working paper reported on DHIS2 implementation experiences in Bangladesh [16]. Several success stories were highlighted including technical advancement in DHIS2 software. According to the UNICEF paper, the proper functioning of DHIS2 could not be achieved thus far due to crucial health system-level challenges [16]. Health system personal were lacking accountability and there was a shortage of human resource availability for data collection and analysis. The culture of using DHIS2 data was limited both within the health ministry and across other ministries [16]. However, global evidence suggests that interdisciplinary coordination is essential for proper functioning of health information technology (IT) [17]. When appropriately designed, digital health technology has proven beneficial in improving the quality of health care through data-driven decisions [17]. Conversely, inappropriate IT design can cause poor human-computer interaction and potential time loss with staff demoralized towards the system [17]. Periodic evaluations and user feedback are vital to sustain digital technology within a health system [18].

With regard to users’ experience with DHIS2 implementation, we lack a clear understanding of the deterrents and enablers of DHIS2 utilization [14]. This information gap has been observed all over the Southeast Asia region [8]. To address this gap, we conducted a qualitative study to explore the perceptions and experiences of different levels of DHIS2 users with implementing DHIS2 to collect, analyze, and use data. We focused on the acceptance of DHIS2 for reproductive, maternal, and newborn health data collection at different levels under DGHS along with the challenges and facilitators of DHIS2 implementation. We aimed to generate evidence for recommendations to strengthen HMIS operations and ameliorate RMNCAH health outcomes in Bangladesh. This subjective knowledge can guide policy makers to plan for future modifications to make DHIS2 more functional.

## Methods

### Study Design & Setting

This qualitative research followed a grounded theory approach [19] at every step, from sample selection to data collection and analysis. The study sites were selected based on the variation in indicator performance among districts according to data retrieved from DHIS2. To choose the study districts, we considered 10 different data sets related to RMNCAH services coming from all 64 administrative districts of Bangladesh for the last one-year period (June 2016 to June 2017). We used the reporting time indicator to track how timely districts report their data. Two stages of screening were performed, first across divisions and then districts. Data from the last 12 months’ performance showed that Khulna was the highest-performing division while Chittagong was the lowest (Additional file 4). Consequently, Jessore district was the highest performing within the Khulna division and Brahmanbaria district was the lowest performing in the Chittagong division. (Additional file 5) shows the districts’ performance within the selected divisions, with panel A representing Khulna division and panel B representing Chittagong division.) We assumed that certain local-level factors like management structures, human resource availability, staff skills, and training might impact workers’ perceptions and experiences with using DHIS2. Keeping this in mind, two upazilas (i.e., administrative unit) from each district were purposely selected. This enabled us to capture local-level influences on DHIS2 use, particularly the differences observed in management and operations at the local level.

### Study participants

Forty-seven stakeholders from all levels of the health system were selected to gather details about experiences with using DHIS2 at each level. The community-level study participants were community health care providers (CHCPs), nurses, health inspectors, upazila statisticians, and upazila health and family planning officers (UHFPOs). The district-level study participants were civil surgeons and district statisticians. The assistant chief of MIS was involved at the division level, and from the national level, system analysts and program managers under MIS directorates and some donor representatives (e.g., monitoring officers, IT programmers) were interviewed. The inclusion criteria was willingness to participate in the study. The exclusion criterion were unwillingness to participate, and those who had been working in the specified sector for fewer than 6 months.

### Data collection methods

The data collection methods were directed by the research objective. Primary data collection methods were in-depth interviews (IDIs), focus group discussions (FGDs), and key informant interviews (KIIs). We collected data from September 2017 through September 2018.

Before initiating data collection, we pretested the interview and FGD guides several times to establish tool validity and reliability. The final guidelines for data collection are added as Additional file 6. The IDIs were conducted with multi-level field staffs: CHCPs, upazila statisticians, nurses, and health inspectors. Study participants for IDIs were selected based on convenient sampling. Emergent questions and reflections from the IDIs were also discussed during the FGDs. Each FGD was comprised of six to seven purposively selected participants. The key informants were categorized into three subgroups at three different levels: health managers (UHFPOs and civil surgeons), HMIS experts (system analysts), IT programmers), and key decision makers (assistant chief MIS-DGHS, program managers from MIS directorates, and divisional focal persons from development partners). We followed a purposive sampling strategy to ensure participation from each stakeholder group. The snowball sampling technique was also used to identify key personal to be interviewed and to ensure rich data collection. An interview time was selected according to participants’ convenience, mostly during after-work hours. A relatively quiet room within the office premises was selected for the interview. Only the interviewer, interviewee, and note taker were present during all face-to-face interview sessions. The interviews lasted from 45 min to 2 hours. All interviews started with a brief introduction of the study objectives, introducing the interviewer, and reading the privacy declaration form. None of the interviewers were previously known to the interviewees and the reason for interrogation was stated as general interest in the research topics. Written informed consent was taken from all the participants in an anonymous privacy declaration form. The interviews were audio recorded with the participants’ consent. Field notes were taken during the interviews to back-up the audio-recorded data in case of equipment failure. The interview was stopped when we achieved data saturation. Table 1 details the data collection methods and purpose.

**Table 1:** Data collection methods and purpose on perception and experiences with using DHIS2

Data collection method	Study respondents	Number of interviews	Sampling technique	Purpose/Main issues explored
IDI	CHCPs, nurses, health inspectors, upazila statisticians	23	Convenient	Efficiency of record keeping using DHIS2; staff attitudes; reporting status; factors hampering data entry and processing
FGD	District statisticians	2	Purposive	Multiplicative information and knowledge of using DHIS2; cross-checking reflections that emerged during the IDIs
KII	Health managers (UHFPOs, civil surgeons)	5	Purposive, snowball	Role of DHIS 2 technology in improving RMNCAH service delivery; constraining and facilitating factors during DHIS2 implementation; other implementation challenges; scope of improvement of servers and software
	HMIS experts (system analysts and IT programmers)	3		
	Key decision makers (assistant chief MIS-DGHS, program managers from MIS directorates and divisional focal persons from development partners)	3		
Total sample size (N)		*N* = 47		

### Data analysis

The collected data was analyzed by content using a thematic approach. We followed the recommended six staged thematic approach consisting of data familiarization, coding, identification, review, naming the major themes, and writing the final report [20]. Interview transcription, translation, and coding were all an iterative process. As part of data familiarization, the research team met regularly during data transcription and cross-checked confusing data against the recordings. For content analysis, we followed a “directed content analysis” approach, where codes were selected both before and after analysis [21]. A-priori codes were prepared using ATLAS.ti. software based on previous research findings and theory [22]. Coding was done independently by two researchers. Intra-coder reliability was checked before listing it in the final codebook.

**Fig. 1:**
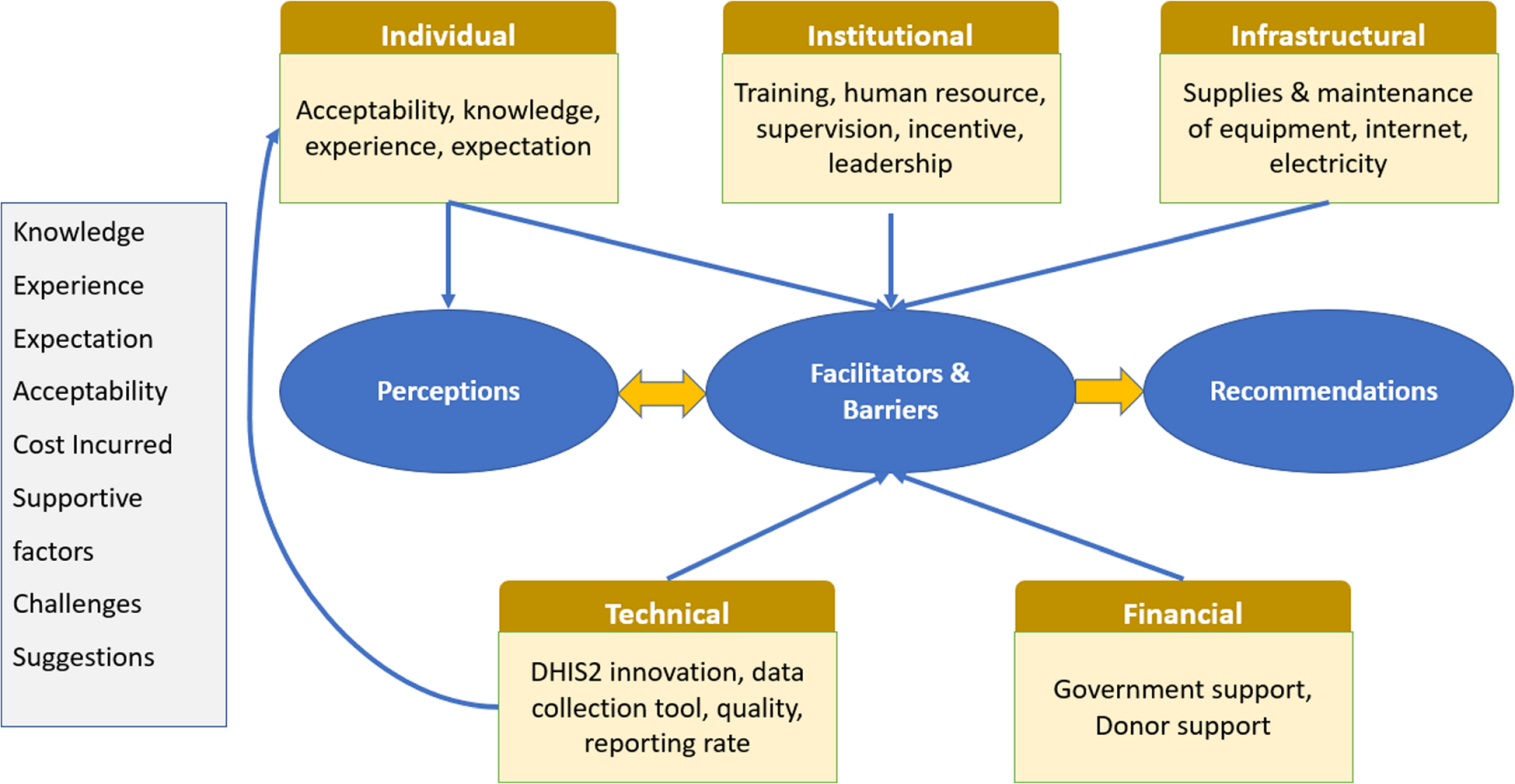
Data structure detailing code plan and core themes of the DHIS2 study in Bangladesh

There were eight initial codes: knowledge, experience, expectation, acceptability, cost incurred, supportive factors, challenges, and suggestions. These codes were condensed to six clusters or subthemes: individual, institutional, infrastructural, financial, technical, and suggestions. Later, we arranged our findings under four key themes that related to the core objectives of our research. The flow of coding to emergent themes is presented in Fig. 1. The sub-theme “individual” captured users’ knowledge, experience, expectations, and their overall acceptability towards an electronic HMIS versus a paper-based system. This sub-theme led to “Key theme 1: Perception”. The code “supportive factors and challenges” was applied when informants talked about positive and negative factors associated with DHIS2 use. Code suggestions (e.g., individual capacity, institutional support, or technical issues related to DHIS2 software) applied when key informants provided their opinions on overcoming challenges or any new suggestions to making the system more functional.

A 32-item checklist to report qualitative research findings known as “Consolidated criteria for reporting qualitative studies (COREQ)” was followed for this research and added as Additional file 7.

The research team translated the coded data into English. We analyzed data from the IDIs, FGDs, and KIIs separately and drew collective inferences from the findings collectively under identified themes [23]. We shared and discussed the findings in a consultative workshop with relevant stakeholders and study participants for data interpretation and validation. The suggestions generated during the workshop were incorporated into the final report under the respective study themes.

## Results

This study identified a set of interrelated concepts across the study informants that influenced the grounded phenomenon, “the overall use of DHIS2”. The study findings were organized under four main key themes:

### Key theme 1

Perceptions of DHIS2. This came from capturing users’ knowledge, experience, expectations, and their overall acceptability towards an electronic HMIS versus a paper-based HMIS.

### Key theme 2

Perceived barriers to implementing DHIS2. These were drawn from the study informants’ discussion of obstacles to DHIS2 use related to individual capacity, institutional support, or technical issues related to DHIS2 software.

### Key theme 3

Perceived facilitators to implementing DHIS2. These were drawn from the study participants’ discussion of positive determinants of DHIS2 use related to individual capacity, institutional support, or technical issues related to DHIS2 software.

### Key theme 4

Recommendations to improve DHIS2 functionality. The study participants shared their suggestions for overcoming challenges or how to make the system more functional.

#### Key theme 1: perceptions of DHIS2

The majority of study participants expressed a strong, positive preference toward using DHIS2 for RMNCAH data collection. They described DHIS2 as a dynamic system that has improved overall medical record keeping and accountability of data reporting from community clinics at the periphery to district-level hospitals.

*Online is a perfect system. Previously I used to collect data in papers, and at the end of the year my office gets full of papers. It was also very difficult to retrieve data from thousands of piled up paper forms. Now, in online, by clicking the date or by name or phone number of the patients, I can easily check the data. I am getting the data collection form even in my mobile, by which I can fill up the form, from any place and at any time! So, it is easier*. — Community health care provider, IDI

The supervisory team perceived that initiating such technology has contributed to instant monitoring, cross-checking of collected data, setting priorities, and making decisions, which was time-consuming with the previous paper-based system. With DHIS2, statisticians are assigned to tabulate the data and share the generated summary reports with district and divisional health managers. Managers observe and flag the gaps in service delivery and note achievements. Findings are discussed at monthly review meetings in the presence of field staff. At these meetings, which are held in each sub-district, district, and divisional health manager’s office, comparisons are made with the previous month, present month, and yearly national targets to track improvements in performance and identify any hindrances to achieving targets. Most respondents, from the community to the national level, identified this review meeting as a platform for RMNCAH-related data observation, monitoring, and instant planning for the coming weeks.

*From DHIS2, along with [the] national scenario, we can see the status of districts and sub-districts, even unions and wards. All the field staff are forwarding data on rate of using contraceptives, maternal death, amount of IUD [intrauterine device] delivered, and number of oral contraceptives supplied*. — Information communication technology focal person, IDI

Additional factors also strongly influenced users’ perception of DHIS2. These were at both the individual and institutional levels. DHIS2 users who were more frequent users and had sufficient training perceived the true need for it. Availability of sufficient technical equipment at the field level, like laptops, desktops, and tablets, made the users more enthusiastic.

The demand for using DHIS2 goes beyond RMNCAH. Key informants who had been involved with DHIS2 since its inception explained that the software is continually maturing. In 2009, when DHIS2 was launched, it was not used for data visualization and decision making because accessing the system was challenging. As soon as DHIS2 introduced the dashboard concept in 2012, it drew the attention of directorates working at the national level, who demanded the platform be used for their own reporting. As a result, the online data entry forms increased from 12 in 2012 to 32 in 2013. The perceived need for DHIS2 is explained in the below quote:

*In 2013, the DHIS2 log-in dashboard became much [more] popular, all users could access it. At that time, 5,000 to 6,000 graphs were made using DHIS2, which eventually increased to 15,000 to 16,000. It means people were trying to use it. To justify my argument, I must say, these graphs were prepared by users from 64 districts, not by a single user. That means people are using it! —* HMIS expert, KII

A few health managers expressed a contrasting view, arguing that staff orientation and adaptation to technology sometimes works as a major obstacle to electronic HMIS implementation. One health manager shared his concern saying that, “*In some places*. *.*. *a complex device, [like a] computer has been handed over to the hand of an old community health worker, hence she cannot use it.”*

While aggregated data are being reported monthly, automated data reporting is not possible within the current DHIS2 system. This makes the data entry process time-consuming and complicated. The field-level workers (i.e., CHCPs) have been maintaining both paper and electronic forms so they can cross-check data from missing reports. Moreover, insufficient understanding of data entry and how to report the RMNCAH indicators leads to unintentional errors in data entry. This ultimately results in more misreporting and less data use.

#### Key theme 2: perceived barriers to implementing DHIS2

##### Technical

Several technical challenges with the DHIS2 platform were highlighted during the KIIs. Absence of an automated data aggregation process increases the possibility of data disparity and errors.

*DHIS2 has a problem. . . . There are [boxes] for entering aggregated data. But, now, it is needed to use the formula. Many of the staff do not understand these formulas. In training sessions, I provide them the formula, explain this using multimedia presentation. Many [field staffs] do not understand it. In several cases, they put the value of one indicator in boxes designated for other indicator.* — IT expert for MIS, KII

Respondents pointed out some technical issues with the data collection forms that should be checked to decrease misreporting and improve efficiency. DHIS2 has the provision to “SKIP” for all indicators, which serves as a source of data incompleteness. Respondents involved in data analysis identified minor issues with the data collection forms that should be checked to decrease misreporting.

*In [the] individual server, first, I put mother's name, her EDD [estimated date of delivery], date of enrolment, and then a box will pop up for gender. There is male, female and transgender. The data is meant to be for the pregnant mother. I don’t understand what the need of gender then? There should be a system that [the] computer would recognize the gender automatically when pregnant woman has been marked. We should not put it manually. Here our field workers are making mistake [s].* — District statistician, FGD

Instead of using unique health identification numbers to track patients, patients’ cell phone numbers are used. However, it is difficult and time-consuming to search the database with a cell phone number. To get around this, CHCPs prefer to enroll follow-up patients as new ones. This raises a data quality issue since repeat clients are identified in the system as new clients. According to the key informants, this has created a gap in the system, as it is not possible to track the health status of a single patient in the existing system during data analysis and visualization. It was suggested that the system could be linked to Bangladesh’s National Identification Database to get a unique identifier.

Updating the data entry forms to facilitate comparisons among data variables is challenging. DHIS2 started with version 2.6 on 2009, which was upgraded, version-by-version, to 2.13 at the end of 2013 to make the system faster. Each time the data entry forms are changed it becomes more difficult to compare the old and new data because the software cannot match the data variables, resulting in invalid findings.

*For those, who are computer literate, for them, a version change is an* “*attraction.”* “*Let us explore, what are the new features?” But our CHCPs do not perceive it in this way. They think, there was a box here in the older version, where did the box goes now with the newer version? They don’t understand, we are trying to make their work easier! It will take some time, to change the culture.* — HMIS expert, KII

Several informants reported that in the existing system, searching for sub-districts is a time-consuming process.

##### Institutional

At the supervisory level, district and sub-district health managers could not find the time to use DHIS2 on a daily basis because they were involved in other activities. Sometimes they avoided using DHIS2 altogether.

*A health manager knows clearly about his district's targets on immunization coverage, or ANC coverage, or even for facility births from their years of experience. So they do not need to open the computer and get into the DHIS2. The mechanism is such; you cannot trap him for this reason.* — Senior programmer, KII

Reporting to DHIS2 is an additional task for the statisticians with other regular administrative duties (e.g., preparing salary sheets, drafting letters). They need to commit an extra hour of work for that. National-level key personnel acknowledged the shortage of statisticians or other staff trained in data analysis. They admitted that in many areas, qualified statisticians had not been recruited. Even so, many statisticians are not proficient in using computer software and do not understand health indicators and data compilation. In many areas, statisticians do not even attend trainings.

*The job description and responsibilities of statistician should be separate. But in many districts there is no designated statistician … . In area “YY,” a ward boy does all the work of a statistician; you cannot expect anything better from him! There should be an assigned person, who will do research [with data].* — District health manager, KII

The RMNCAH data collected by the MIS Division of DGHS is also used by the RMNCAH line directorate of DGHS. However, data retrieval from the DHIS2 platform is not the regular practice for the RMNCAH line directorates; like all other directorates they rely on their own reporting format.

Statisticians reported not receiving any specific training on DHIS2, rather it was a part of computer literacy training. Participants received DHIS2 training manuals, though these were not updated to reflect changes in newer versions of the software and forms. Since DHIS2 was introduced, all the line directorates want to incorporate their relevant indicators to be collected and analyzed through DHIS2 using the same workforce.

*Now everybody wants their data from DHIS2. [The] non-communicable disease division add some indicator [s], RMNCAH add some too. In some cases, the reporting format is also different than the one used by DHIS2. For example, if [the] EmNOC [emergency newborn and obstetric care] reporting format for [the] MIS division and RMNCAH would be [the] same, I can get the report by clicking on DHIS2 data. But [the] EmNOC report for RMNCAH directorates have 27 indicators while it is 25 in [the] DHIS2 database. —* Sub-district statistician, IDI

##### Infrastructure

Although the participants said the number of electronics provided for data collection is sufficient, slow Internet connectivity makes real-time data entry difficult. As one CHCP described:

*At dawn, sometimes the Internet speed is better. In most cases, I enter the data at this time. It happened, I could not report for one week, two weeks, as the speed was slow. With a weak connection, I cannot even log in into the system. —*CHCP, IDI

Providing offline data entry could make things easier. The process of sending broken tablets to the capital city for repairs and transporting them back to the community takes a long time. The majority of respondents reported internet modem shortages as well. In many areas, sub-district and district health managers personally obtained a modem and Wi-Fi router.

#### Key theme 3: facilitators of data collection and analysis with DHIS2

Mandatory quality checks at different tiers have played a significant role in improving data quality. At the data entry level, the system does not allow incorrect data. The system ensures self-validation of data by adopting three approaches: input validation, adding appropriate ranges, and validation rules. When a data operator adds any value that is out of the expected range, they get an error message. Moreover, DHIS2 allows local-level data access and correction before it is reported at the national warehouse. An IT focal person with a medical background was assigned at a sub-district hospital and at a district-level civil surgeon office to check data errors. Consequently, regular monthly feedback meetings are organized at the sub-district level in the presence of field workers from both the health and family planning wings to minimize duplication of RMCAH data. Similarly, monthly feedback meetings are also organized at the district and national levels. The national core MIS committee, chaired by the MIS directorates, meet monthly to get feedback on technical issues and to monitor data coming from all the districts. The key informants greatly appreciated this national-level meeting where government officials, donors, and technical people participate.

A national-level expert shared his experience with checking data validity:

*For example, when we check MMR [maternal mortality ratio], we locate where the ratio is high. Then we review the ratio of that particular district for consecutive months to explore the consistency of data and reporting status, either it was low or high for the previous months. We check all these. Then we send an e-mail, to respective authority, to look into the matter.* — HMIS expert, KII

So far, DHIS2’s performance has been measured from the perspective of timeliness and completeness. The sub-district and districts are evaluated according to their overall reporting rate. A positive competition for service improvement has been nurtured. The best-performing district or division receives recognition from the national level.

*In our monthly meeting we discuss our shortfall; we plan how to improve the reporting rate. We always analyze the data, hence our performance is better!! We have a silent competition with other districts of this division and we do better always and got national award as model district.* — District health manager, KII

International donors strongly support strengthening Bangladesh’s HMIS. They share financial costs with the government for national- and international-level staff training, IT equipment purchases, and other needs. In collaboration with other nongovernmental organizations, like icddr,b, they are providing technical support to the IT programmer for improving the online platform and organizing a training on the DHIS2 manual for staff working at different tiers of the health system. Donor organizations have demonstrated a strong commitment to the successful implementation of DHIS2 by deploying their staff as monitoring officers at each administrative division and ensuring their physical presence and participation during monthly coordination meetings at the divisional and central levels.

*The government has limited capacity and could not develop that capability till now. From the side of development partners, we are giving them that support. If development partners withdraw their support, how will the system run? But the DHIS2 dashboard is already sustainable, and its automatic; staff have training and they can handle it. The government is cordial, and they have sufficient resources, training arrangements, and hardware. In this context, strict monitoring and defined role of staffs are important. In addition, ownership of data is a major concern, many health managers do not own the data.* — HMIS expert from donor, KII

**Fig. 2:**
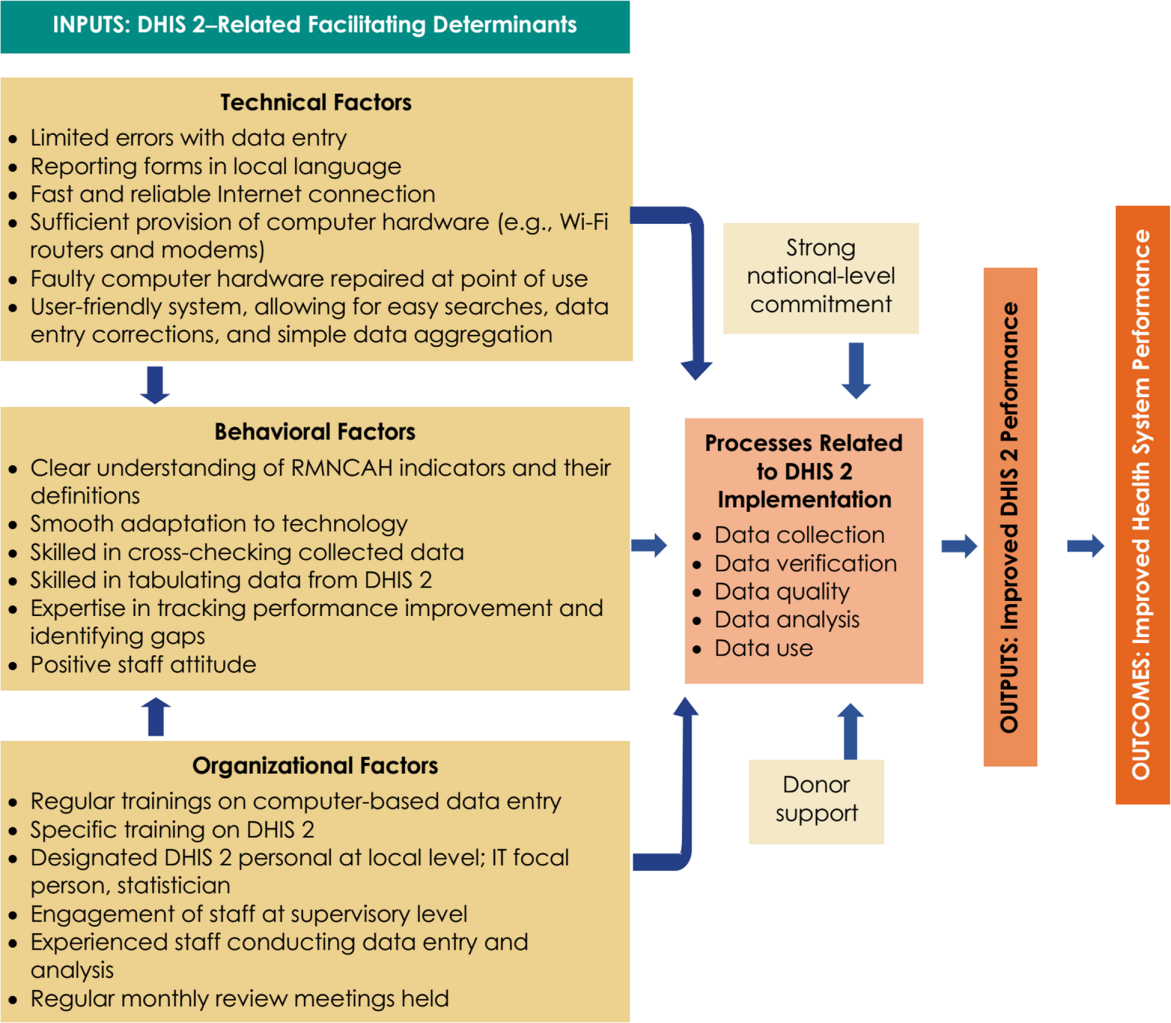
Analytic framework on strengthened DHIS2 in Bangladesh

A monitoring and evaluation framework is used to identify DHIS2-related facilitating factors at all steps ranging from input, process, and outcome (Fig. 2).

#### Key theme 4. Recommendations for strengthening the HMIS to improve RMNCAH outcomes

Based on the study findings, the participants’ major recommendations for strengthening the HMIS to ameliorate RMNCAH outcomes in Bangladesh are elaborated in this section. The DHIS2 platform should be programmed to generate automated data for specific RMNCAH indicators. A pop-up box with the indicator definition, calculation (if applicable), and any possible disaggregation should be included. This will provide instant help to the CHCPs and standardize data collection. The software should be translated into Bangla (the local language) to help create a clear understanding of instructions and RMNCAH indicators. An online dashboard should be installed in the platform where instant RMNCAH-related reporting and performance status updates can be exhibited automatically at the sub-district and district levels. Statisticians should be informed in advance about software updates and notified of specific changes so they can prepare the CHCPs.

Data collection forms should be simplified to ease the data collection process and data reporting. Creating unique health identification numbers for patients and issuing individual health cards will decrease the time spent on data entry and help mitigate data duplication. Since the system will contain clients’ contact information, statisticians can verify the collected data through random phone calls. A geographic information system should be installed in CHCPs’ electronic devices used for data collection to track providers’ movement. Users should be able to enter data into DHIS2 daily, as aggregated data increases the risk of errors and compromises data quality.

Since DHIS2 is used at different levels of the health system, the DHIS2 training curriculum should be tailored to the needs of health professionals working at different levels. The IDIs and FGDs revealed a need for separate training sessions on medical terminology for community- and sub-district-level staff. After every update to the software or data collection forms, refresher trainings should be organized to improve staff knowledge and efficiency. A standardized training curriculum and tools are also needed. Furthermore, soft copies of training manuals should be shared with staff via e-mail so they can be easily updated and disseminated.

Along with a statistician, another staff member should be trained in data compilation and analysis to complement the statistician’s work and support the statistician in his/her absence. A separate MIS unit can be formed comprising, at a minimum, a statistician and a supporting staff member who will be assigned to perform all MIS-related tasks only. Sub-district and district health managers should be more involved in data reporting and analysis to develop ownership and a regular practice of using DHIS2.

Computers and other electronic devices for data collection should be repaired at the local level to save money and time. Providing CHCPs with an Internet data subscription can ensure timely reporting. The number of modems at the sub-district and district levels should be increased, and each municipality should have its own dedicated laptop for the statisticians to use to ensure timely reporting.

The country would benefit from a national e-health strategy and implementation framework to facilitate a culture of DHIS2 use for planning, setting priorities, and decision making among different stakeholder groups. This strategy should include how the country intends to provide the resources to fund DHIS2’s long-term sustainability when donor support is no longer available.

## Discussion

We observed a strong, positive attitude toward a digitized e-health system among our study informants. The parallel actions taken from the MIS directorates could make nationwide implementation of DHIS2 possible in Bangladesh. As facilitating factors, informants highlighted some of the unique initiatives taken by the Bangladesh health system. Among them, the presence of an IT focal person at the peripheral level health facilities, the option for both online and offline data entry, and adding a DHIS2 dashboard to the online platform were commonly mentioned factors. The health mangers shared that the dashboard makes the system more user-friendly for local-level planning. Similarly, DHIS2 users from other countries opined that data collected through DHIS2 needs to be analyzed and used at more frequent intervals. DHIS2 dashboards were introduced for data visualization on an everyday basis. They mentioned that this graphical presentation helped them to identify the required inputs to overcome service gaps [24–26].

Mandatory data quality checks and regular monthly coordination meeting at different tiers of the health system, with active participation from all levels of key stakeholders, were other positive influencing factors in our study. The importance of data review meetings to strengthen the HIS has been highlighted in other studies too [12, 27]. However, some key informants mentioned that data quality was still compromised due to problems with the data collection tools. Moreover, in the absence of an automated system, errors happen while summing up the monthly report. The importance of an automated system in DHIS2 has been mentioned in studies from Africa. They found that the automated reporting system minimized reporting time and increased the completeness and accuracy of MNCH data in their contexts [28, 29].

Although data accuracy and completeness remain an issue, users were encouraged to use DHIS2. To increase the use of DHIS2, Bangladesh’s MIS division has started to announce and reward the best-performing district on an annual basis. While measuring district performance, timeliness to provide monthly reports is prioritized. This is expected to enhance positive competition among DHIS2 users. Other studies also highlighted the importance of incentives for increasing DHIS2 use. Researchers mentioned that adding incentives for accurate and timely reporting could bring a positive mindset change among field-level health workers in their settings [10, 27, 30].

The barriers to implementing an electronic HMIS mentioned in this study are similar to those reported in other developing countries: inadequate human resources, frequent power outages, low Internet connectivity, and a poor culture of using data for decision making [25, 31–34]. Some older field staff are still struggling with the mindset change – trusting electronic record keeping during the move from paper-based data collection and appreciating the usefulness of collected data. In this regard, another study highlighted that time is needed to allow community health workers to adapt and increase their computer literacy. Their suggestion for overcoming this barrier was to organize onsite supportive supervision and provide trouble shooting at the district level [11, 35]. Another suggestion from a successful DHIS2 implementer country was to ensure data ownership. Data ownership enables field-level workers to understand the purpose of data collection and how the information will be used rather than considering it an administrative burden [11].

The data collected under DHIS2 was considered comprehensive in our study. In addition to collecting RMNCAH health indicators, the system captures human resources, medicine, and logistics data. This data availability allows the health system to address broader contextual factors like human resource shortages or stock outs, which can act as hindrances to achieving high-quality RMNCAH data [15, 29].

We identified a training need for field-level staff. The training was found inappropriate to meet the demand of all levels of DHIS2 users. Our study participants perceived that the DHIS2 training curriculum should be tailored to the needs of health professionals working at different levels. They suggested separate training sessions on medical terminology for the community and sub-district-level staff. They also demanded refresher trainings, particularly after every update to the DHIS2 software or after any changes made to the data collection forms. In line with our study, insufficient attention on training needs has also been identified as a major challenge with DHIS2 use in Ghana [32] and in Zanzibar [36]. To overcome this challenge, some effective strategies were found in Kenya. They introduced “training of trainers” and “on-the-job trainings” for DHIS2 users [37]. Moreover, the international DHIS2 academy has provided extensive support across the world to address any training needs [38] . Considering the vast utility of DHIS2, the University of Colombo in Sri Lanka has introduced DHIS2 in their master’s in science course on biomedical informatics. The new graduates are expecting to contribute to the country’s progress towards DHIS2 use [39].

Although the study participants felt the data collected under DHIS 2 were comprehensive, the system lacks critical information, such as family planning statistics. Because the DGFP MIS is not connected to DHIS2, data collected under the DGFP cannot be accessed and analyzed with DHIS2. This lack of synchronization creates parallel reporting systems [40]. The study participants highlighted this duplicity in reporting and mentioned the difficulty of managing multiple forms and reporting the same RMNCAH indicators in different formats for different stakeholders. However, to mitigate this data duplication issue, a pilot project funded by the United States Agency for International Development launched in 20 sub-districts of Tangail and Habiganj in Bangladesh. Every eligible mother and under-five child at the community level has received a health card with a unique identification number that allows the system to retrieve and monitor their MNCH service uptake data at any service delivery point [41]. The outcome of this project will support our understanding of the feasibility of HMIS’s unification of the DGHS and DGFP.

Another challenge with getting comprehensive RMCAH data lies in that the majority of urban health facilities in Bangladesh are not reporting in DHIS2. The urban health system is dominated by the private sector, which is not accountable for monthly reporting to DHIS2. A similar situation has been observed in many African countries where private health facilities are not reporting through routine DHIS2 reporting [35]. A fragmented HMIS, with duplicate data collection, lack of data sharing, and incomplete data collection, are common HIS problems in the majority of lower-income countries [42].

The national-level key informants mentioned that DHIS2 implementation in Bangladesh is largely dependent on international donors. To ensure sustainability, they suggest that the country adopts its own strategy to self-finance the HIS. They also suggest the need for a national e-health strategy for better interdisciplinary actions. Evidence from other successful DHIS2 implementing countries has shown that capacity building of IT staff and funding generated through public-private partnerships were successful strategies for ameliorating donor dependency [3, 27].

### Limitation

A limitation to our study is that we did not explore the views of staff in other directorates which are closely linked with RMNCAH data collection and use because of budgetary and time constraints. It would be worth interviewing national-level key personal from the DGFP and RMNCAH line directorate of DGHS. They are the key personal involved in national-level priority setting for meeting the national and global agenda on MNCH outcomes and their practical experience with using DHIS2 data could add a complete understanding of DHIS2 functionality. We tried to compensate for this shortcoming by interviewing district-level managers and major donor representatives who are working both for electronic MIS and MNCH service improvement in Bangladesh.

## Conclusions

Our study found that most study participants exhibited a positive attitude toward an electronic HIS. Although DHIS2 has become the data repository for different health data, multiple reporting formats for different stakeholders, in addition to the ongoing reporting requirements, negatively impacts the workload of field-level health workers. The exclusion of data from Bangladesh’s large private health sector is a hindrance to getting a complete picture of the country’s RMNCAH status. Slow Internet connectivity, some health workers’ defensive attitude toward an electronic system, and limited use of data for local-level decision making prevent the successful implementation of DHIS2. We recommend periodic refresher trainings to increase staff confidence in computer literacy. A national e-health strategy and implementation framework, as recommended by key stakeholders, will outline how the country will fund the sustainability of DHIS2 and facilitate a culture of data use for planning, setting priorities, and decision making.

## Supplementary information

**Additional file 1.** Common RMNCAH indicators retrieved by the DHIS2 under DGHS

**Additional file 2.** HMIS data flow under Director General of Health, Bangladesh

**Additional file 3.** HMIS data flow under Director General of Family Planning, Bangladesh

**Additional file 4.** Performance of DHIS2 across division

**Additional file 5.** Performance of DHIS2 across Khulna division (Panel A) and across Chittagong division (Panel B)

**Additional file 6.** Interview guidelines

**Additional file 7.** Study quality assessment checklist (COREQ checklist)

## Data Availability

Data supporting this study findings will be available from the Research administration of icddr,b with reasonable request of anonymous data. Please email to director research administration of icddr,b for further data request.

## Abbreviations

ANC: Antenatal care
CHCP: Community healthcare provider
DGHS: Directorate General of Health Services
DGFP: Directorate General of Family Planning
e-health: electronic health
FGD: Focus group discussion
HIS: Health information system(s)
HMIS: Health management information system(s)
IDI: In-depth interview
IT: Information technology
KII: Key informant interview
MIS: Management information system(s)
MOHFW: Ministry of Health and Family Welfare
MNCH: Maternal, newborn, and child health
PNC: Postnatal care
RMNCAH: Reproductive, maternal, newborn, child, and adolescent health
UHFPO: Upazila health and family planning officer
WHO: World Health Organization
