# Supplementary material for: Perceptions and experiences with district health information system software to collect and utilize health data in Bangladesh: a qualitative exploratory study
Source: BMC Health Serv Res. 2020 May 26;20:465. doi: 10.1186/s12913-020-05322-2 (PMC7249629; doi:10.1186/s12913-020-05322-2)
Supplement: Supplementary file 1 — Additional file 1. Common RMNCAH indicators retrieved by the DHIS2 under DGHS [file 12913_2020_5322_MOESM1_ESM.docx]

Additional Table 1: Commonly reported RMNCAH indicators in DHIS 2 under DGHS

| Type of Data | Related Indicators* |
| --- | --- |
| Emergency obstetric care | # of ANC^a^ visits |
|  | # of PNC^b^ visits |
|  | Type of delivery (disaggregated by normal or caesarean section) |
|  | Skilled delivery rate |
|  | Pregnancy complications |
| Immunization | # of pregnant women receiving tetanus-toxoid vaccine |
|  | # of children under five receiving scheduled vaccines, by type |
| Integrated management of childhood illness | # of integrated management of childhood illness pneumonia cases treated at outdoor & indoor department of health facility |
| Mortality | # of maternal deaths |
|  | # of stillborn deaths |
|  | # of neonatal deaths |
|  | # of under-five deaths, by month |
| Nutrition status of women and children under five | # of underweight & stunting cases treated for children under five |
|  | # of anemic mothers receiving iron folate supplements |
| Family planning | # of short- and long-term contraceptives distributed |
|  | # of referrals given for long-acting family planning methods |
| Health education sessions | # of health education sessions held on family planning methods |
|  | # of health education sessions held for pregnant women on nutrition, danger signs of pregnancy, etc. |
| Other reproductive health | # number of vaginal inspection assessments |
|  | # of cervical cancer screenings done at health facility |
| Human resources | # of sanctioned (i.e., allocated) posts |
|  | # of available posts |
| Hospital logistics | # of amenities and hospital equipment |
|  | % of equipment functioning |

*Not inclusive list ^a^ANC: Antenatal Care ^b^PNC Post natal care
