# Supplementary figures and images for: Perceptions and experiences with district health information system software to collect and utilize health data in Bangladesh: a qualitative exploratory study
Source: BMC Health Serv Res. 2020 May 26;20:465. doi: 10.1186/s12913-020-05322-2 (PMC7249629; doi:10.1186/s12913-020-05322-2)

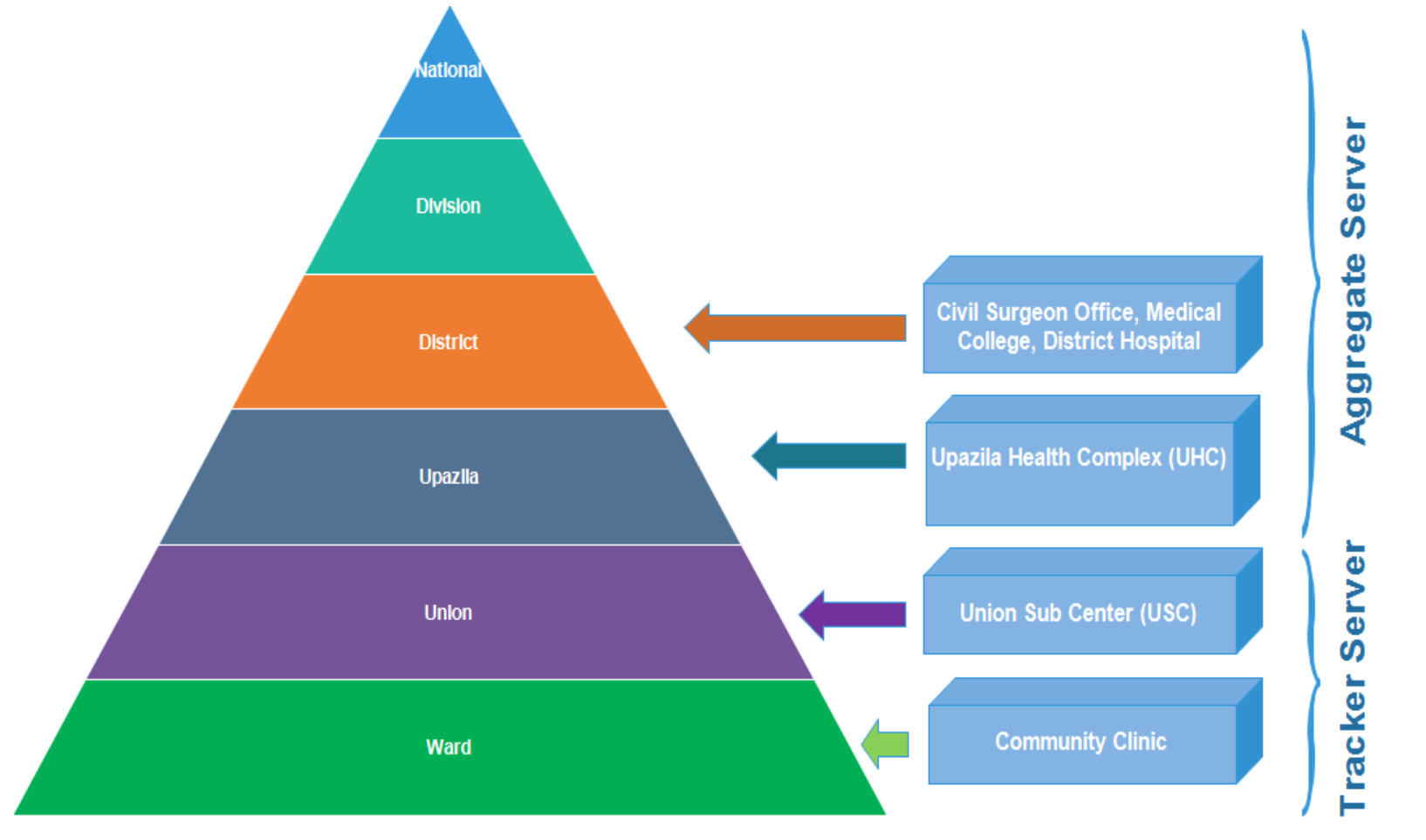

Supplement: Supplementary file 2 — Additional file 2. HMIS data flow under Director General of Health, Bangladesh [file 12913_2020_5322_MOESM2_ESM.png]

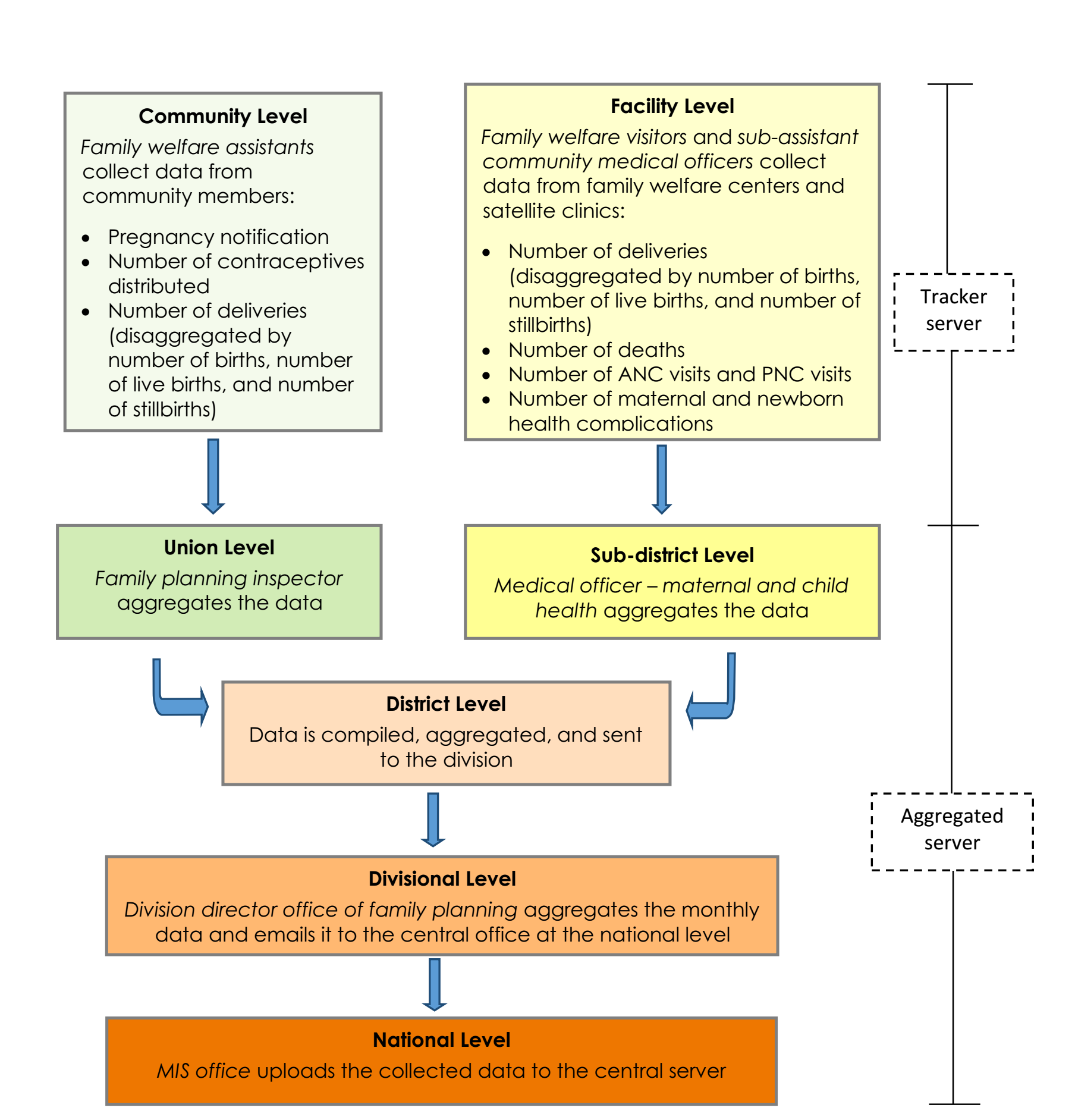

Supplement: Supplementary file 3 — Additional file 3. HMIS data flow under Director General of Family Planning, Bangladesh [file 12913_2020_5322_MOESM3_ESM.png]

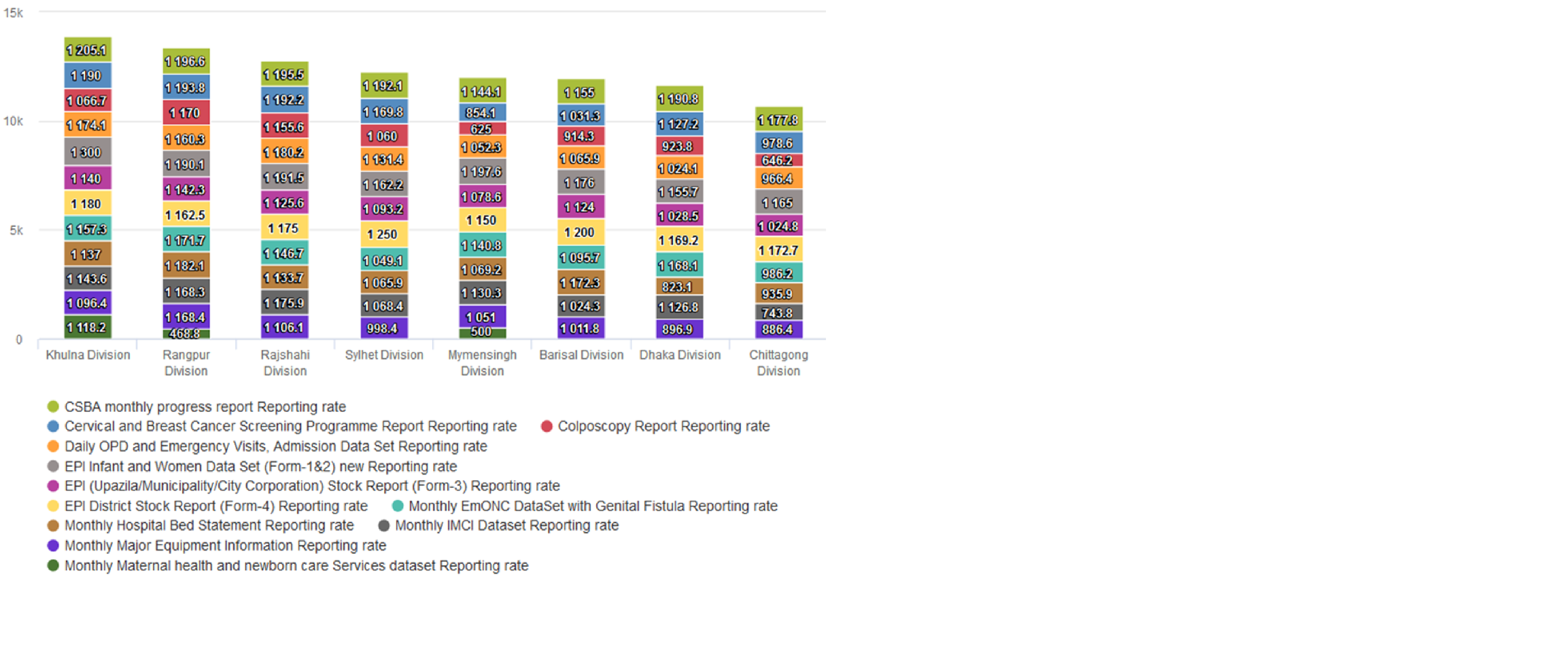

Supplement: Supplementary file 4 — Additional file 4. Performance of DHIS2 across division [file 12913_2020_5322_MOESM4_ESM.png]

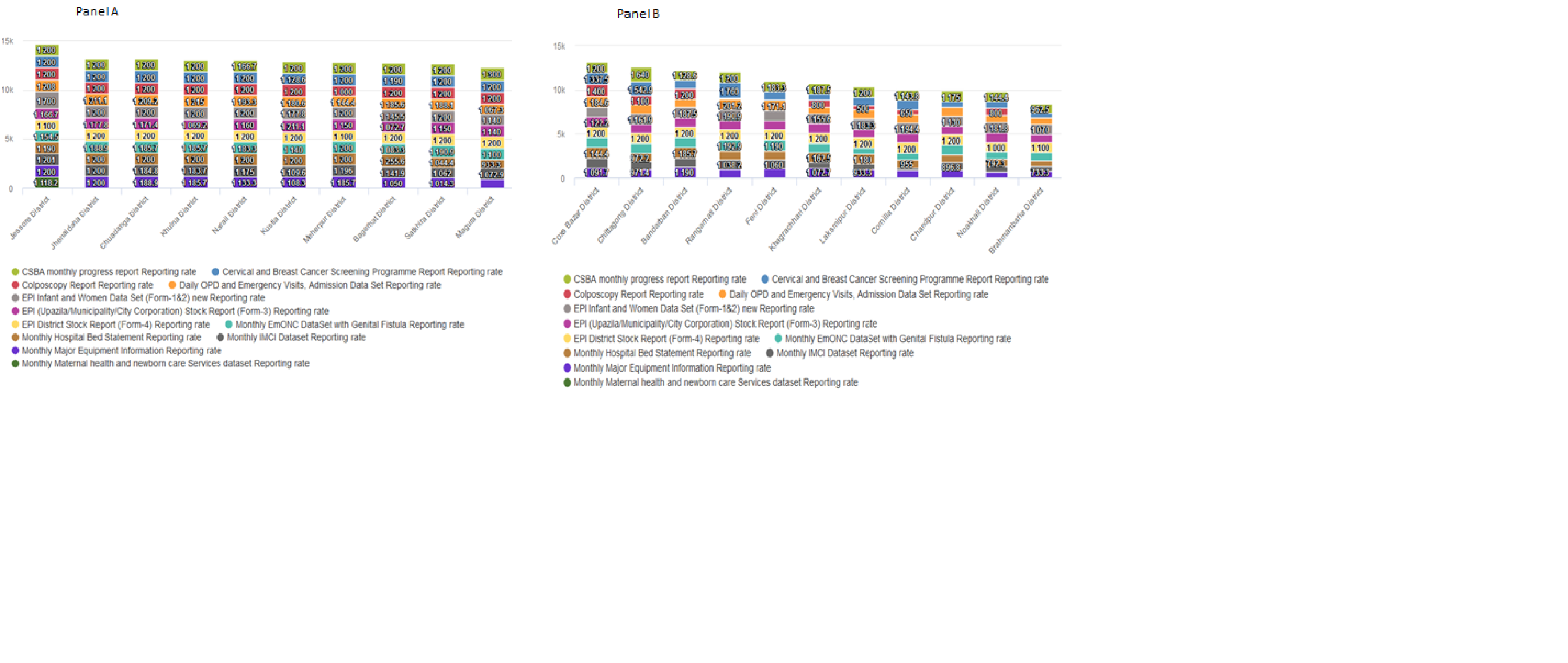

Supplement: Supplementary file 5 — Additional file 5. Performance of DHIS2 across Khulna division (Panel A) and across Chittagong division (Panel B) [file 12913_2020_5322_MOESM5_ESM.png]
