# Supplementary material for: Perceptions and experiences with district health information system software to collect and utilize health data in Bangladesh: a qualitative exploratory study
Source: BMC Health Serv Res. 2020 May 26;20:465. doi: 10.1186/s12913-020-05322-2 (PMC7249629; doi:10.1186/s12913-020-05322-2)
Supplement: Supplementary file 6 — Additional file 6. Interview guidelines [file 12913_2020_5322_MOESM6_ESM.pdf]

Appendix 6.1: In depth Interview Guideline

Protocol Title: Perceptions and experiences on implementing technology (DHIS2 software) to collect and utilize the reproductive, maternal and neonatal health related data in Bangladesh
Protocol number: 17086

Note:
Start by introducing yourself, the purpose and the expected time (1 hour).
If agreeable to interview, proceed to the consent form
Request to record interview and make sure the tape recorder is switched on to the start of the interview. If denied, please make note.

1. Current Position

2. Age

3. Educational background

4. How have you been involved in using DHIS2 software for data collection?

- Since when?
- What is your role?
- **Make notes on:** Years of involvement in using DHIS2 software

5. Let us brain storm and list freely list all tasks required to accomplish using DHIS2 (probe on

- What are the major tasks/type of tasks? & what type of data you need to enter
- Does anyone assist you in this regard? And please mention how much helpful this assistance is?

6. Please share your experience of data collection and entry using DHIS2 with us.

- How frequently do you use it?
- How do you use it?

7. From your experience, how do you perceive the data collection process? Please share your thoughts with us. (Aim: to know the practical experience with data collection, compilation and preparing report)

- Ask about data collection forms (difficult/easy & why he perceive so)
- Compilation: Probe on any difficulty on gathering required data, any need of maintaining hard copy of registrar (advantage or disadvantage of it)
- Reporting: does it useful to report all required RMNACH indicators?
- Others: language barrier, conflict with usual clinical task

8. How confident do you feel when you give the data entry?

- Please rank on a 5-point scale: (Strongly agree) 1-2-3-4-5 (Strongly disagree)
- Why did you rank yourself in that position?

9. Have you ever faced any challenges in using DHIS2 for your work purpose?

- Please mention about problems, that you are frequently facing
- How did manage/overcome those problems?
- Challenges that could be solved at low cost immediately

10. Have you ever faced any difficulty for data entry/forwarding the data to your supervisor?
11. How does your supervisor share the findings from DHIS2 dashboard in the monthly meetings?

    What are the common feedbacks for data collections and entry?

12. Does your supervisor provide you any individual/group feedback?

    - What are the suggestions, your supervisor provided you to improve your performance?
    - Do you find those helpful?

13. In your opinion, what are the common mistakes/confusions for data entry?

    How those common mistakes/confusions could be solved?

14. Have you received any training/attended any workshop on DHIS2?

- When?
- Have you received any training manual/reading materials?
- Any feedback on training (e.g. course content, facilitation)
- Future need for training (e.g. frequency of training, additional training for the staffs who are trained already)

15. What is the overall strengths/weakness in using DHIS2 software for data collection in your locality, specifically?

Final observations

16. What could be done to make the DHIS2 software more usable and understandable at community level?

- What kind of support do you need?
- Scope of improvement

17. Can you suggest names of other actors who might be interested in participating in this study? *[If yes]* Would it be okay if we used your name when we contact these people to say that you recommended them to us? *[Collect names and email addresses of recommended individuals*

Finish the Interview by thanking the participant.

Appendix 6.2: Focus Group Discussion (FGD) Guide

Protocol Title: Perceptions and experiences on implementing technology (DHIS2 software) to collect and utilize the reproductive, maternal and neonatal health related data in Bangladesh
Protocol number: 17086

Note:
Start by introducing yourself, the purpose and the expected time (1 hour).
If agreeable to participate in the discussion, proceed to the consent form to every participant.
Request to record FGD and make sure the tape recorder is switched on to the start of the discussion.
Request to listen to others opinions and to be respectful to one another.

Introduction with the participants

1. How have you been involved in using DHIS2 software for data collection
- Since when?
- What is your role?
- **Make notes on:** Years of involvement in using DHIS2 software for each participant
2. Let us brain storm and list freely all tasks required to accomplish using DHIS2 [*Please use a flip paper*]
- What are the major tasks/type of tasks?
- Does anyone assist you in this regard?
3. Where would you put yourself, in accomplishing your task using DHIS2? [*Please use the same flip paper with enlisted tasks and paste green stickers for easier tasks and red sticker for difficult tasks*]
   Please ask for what made these tasks more or less difficult

4. Please share your experience of data collection and entry using DHIS2 with us.
   - How frequently do you use it?
   - How do you use it?

5. From your experience, how do you perceive the data collection process? Please share your thoughts with us.
- Do you think the data collection forms are difficult to understand?
- Do you think the commands for data entry are critical?
- Others: language barrier, limited logistic support

6. How confident do you feel when you give the data entry?
- Please rank on a 5-point scale: 0-1-2-3-4-5
- Why did you rank yourself in that position?

7. Have you ever faced any challenges in using DHIS2 for your work purpose?

- Please mention about problems, that you are frequently facing
- How did manage/overcome those problems?
- Challenges that could be solved at low cost immediately

8. Have you ever faced any difficulty for data entry/forwarding the data to your supervisor?
9. How does your supervisor share the findings from DHIS2 dashboard in the monthly meetings?

- What are the common feedbacks for data collections and entry?

10. Does your supervisor provide you any individual/group feedback?

- What are the suggestions, your supervisor provided you to improve your performance?
- Do you find those helpful?

11.. In your opinion, what are the common mistakes/confusions for data entry?

- How those common mistakes/confusions could be solved?

12. Have you received any training/attended any workshop on DHIS2?

- When?
- Have you received any training manual/reading materials?
- Any feedback on training (e.g. course content, facilitation)
- Future need for training (e.g. frequency of training, additional training for the staffs who are trained already)

13. What is the overall strengths/weakness in using DHIS2 software for data collection in your locality, specifically?

Final observations

14. . What could be done to make the DHIS2 software more usable and understandable at community level?

- What kind of support do you need?
- Scope of improvement

15. Can you suggest names of other actors who might be interested in participating in this study? *[If yes]* Would it be okay if we used your name when we contact these people to say that you recommended them to us? *[Collect names and email addresses of recommended individuals*

Finish the FGD by thanking the participants.

Appendix 6.3: Key Informant Interview Guideline

Protocol Title: Perceptions and experiences on implementing technology (DHIS2 software) to collect and utilize the reproductive, maternal and neonatal health related data in Bangladesh
Protocol number: 17086

Note:
Start by introducing yourself, the purpose and the expected time (1 hour).
If agreeable to interview, proceed to the consent form
Request to record interview and make sure the tape recorder is switched on to the start of the interview. If denied, please make note.

1. Name

2. Current Position

3. **How have you been involved in using DHIS2 software?**
   - Since when?
   - What did/do you do using the DHIS2 (monitoring of data collection and entry/ data analysis/ data utilization for decision making)
   - **Make notes on:** Years of involvement in using DHIS2 software
4. **Let us brain storm and list freely all tasks required to accomplish using DHIS2**
   - What are the major tasks?
   - How does DHIS2 software help you in this regard?
   - To carry out your roles and responsibilities using DHIS2 software, what are the competencies needed?
   - Does anyone assist you in this regard?
   - If YES, how do the staffs support you?
5. **Do you have sufficient workforce to assist you?**
   - How do you manage, when staffs take leave of absence?
   - Does it affect your work?
6. **Have you received any training/attended any workshop on DHIS2?**
   - When?
   - Have you received any training manual/reading materials?
   - Any feedback on training (e.g. course content, facilitation)
   - Future need for training (e.g. frequency of training, additional training for the staffs who are trained already)
7. **Please share your experience of using DHIS2 with us.**
   - How frequently do you use it?

8. **How confident do you feel when you use DHIS2?**

   Please rank on a 5-point scale: (Strongly agree) 1-2-3-4-5 (Strongly disagree)

9. **Why did you rank yourself in that position?**

10. **How do you perceive your competencies for monitoring the data collection/analysis/utilization process?**

Please share your thoughts with us.

- Please rank on a 5-point scale: (Strongly agree) 1-2-3-4-5 (Strongly disagree)
- Why did you rank yourself in that position?

11. In your opinion, how far do you think DHIS2 dashboard is understandable and usable for your work purpose?

- Do you think the data collection forms are difficult to understand?
- Do you think the commands for data analysis and utilization are critical?
- Others: Language barrier

12. Have you ever faced any challenges in using DHIS2 at your work?

- Please mention about problems, that you/your staffs frequently face (e.g., logistical challenges)
- How did/do manage/overcome those problems?
- Challenges that could be solved at low cost

13. How do you share the findings from dashboard in the monthly meetings?

14. To what extent DHIS2 helps you in planning, monitoring and decision making?

- Can you please give us one or two examples?
- Any challenges faced, in doing so

15. How do you encourage/motivate your staffs to use DHIS2?

16. Do you know anyone who uses DHIS2 frequently?

How do you know?

17. Do you share the findings from DHIS2 dashboard in the monthly meetings?

How do you use the findings for strategic planning?

18. What is the overall strengths/weakness in DHIS2 software utilization in your locality, specifically?

Final observations

19. Where would you put yourself in utilizing DHIS2 software?

Please rank on a 5-point scale: (Strongly agree) 1-2-3-4-5 (Strongly disagree)

20. Why did you rank yourself in that position?

21. What could be done to increase the usage of DHIS2 software at your office/among your staffs?

- What kind of support do you need?
- Scope of improvement

22. Can you suggest names of other actors who might be interested in participating in this study? ***[If** yes]* Would it be okay if we used your name when we contact these people to say that you recommended them to us? *[Collect names and email addresses of recommended individuals.]*

Finish the Interview by thanking the participation
