# Supplementary material for: Perceptions and experiences with district health information system software to collect and utilize health data in Bangladesh: a qualitative exploratory study
Source: BMC Health Serv Res. 2020 May 26;20:465. doi: 10.1186/s12913-020-05322-2 (PMC7249629; doi:10.1186/s12913-020-05322-2)
Supplement: Supplementary file 7 — Additional file 7. Study quality assessment checklist (COREQ checklist) [file 12913_2020_5322_MOESM7_ESM.pdf]

Table: Consolidated criteria for reporting qualitative studies (COREQ): 32-item checklist

No	Item	Guide questions/description	Reference
**Domain 1: Research team and reflexivity**			
Personal Characteristics			
1.	Interviewer/facilitator	Which author/s conducted the interview or focus group?	TB & FAK
2.	Credentials	What were the researcher's credentials? *E.g. PhD, MD*	Title page: line 6- 14<br>PhD (IA,AB)<br>MD(TB,AR,JF)<br>MPH (TB,SMK,AR,JF,IA)<br>MSS (FAK)<br>MSc (SI, MP)
3.	Occupation	What was their occupation at the time of the study?	Title page: line 6- 14
4.	Gender	Was the researcher male or female?	Female(TB,SMK,AB,JF,FAK)<br>Male (IA,AR,MP,SI)
5.	Experience and training	What experience or training did the researcher have?	Title page: line 6- 14<br>MPH: (TB,SMK,AR,JF,IA,AB)<br>Masters in Social Science : FAK<br>Training on qualitative data collection and analysis: TB,FAK & SMK

No	Item	Guide questions/description	Reference
Relationship with participants			
6.	Relationship established	Was a relationship established prior to study commencement?	Data collection methods line 124-125
7.	Participant knowledge of the interviewer	What did the participants know about the researcher? e.*g. personal goals, reasons for doing the research*	Data collection methods line 125-126
8.	Interviewer characteristics	What characteristics were reported about the interviewer/facilitator? e.g. *Bias, assumptions, reasons and interests in the research topic*	Data collection methods line 126-127
**Domain 2: study design**			
Theoretical framework			
9.	Methodological orientation and Theory	What methodological orientation was stated to underpin the study? *e.g. grounded theory, discourse analysis, ethnography, phenomenology, content analysis*	Data analysis line 134
Participant selection			
10.	Sampling	How were participants selected? *e.g. purposive, convenience, consecutive, snowball*	Data collection methods line 113,115 & 118 Convenient for IDI , purposive for FGD & KII
11.	Method of approach	How were participants approached? e.*g. face-to-face, telephone, mail, email*	Data collection methods Line 123 (Face to face)

No	Item	Guide questions/description	Reference
12.	Sample size	How many participants were in the study?	Study participants line 93 & table:1 total sample size 566
13.	Non-participation	How many people refused to participate or dropped out? Reasons?	None
Setting			
14.	Setting of data collection	Where was the data collected? e.*g. home, clinic, workplace*	Data collection methods line 122 (office premises, after office hour in a quiet room)
15.	Presence of non-participants	Was anyone else present besides the participants and researchers?	Data collection methods line 122-123 (Interviewer ,interviewee & note taker)
16.	Description of sample	What are the important characteristics of the sample? *e.g. demographic data, date*	Study participants line 96 to 103
Data collection			
17.	Interview guide	Were questions, prompts, guides provided by the authors? Was it pilot tested?	Data collection methods line 110-111
18.	Repeat interviews	Were repeat interviews carried out? If yes, how many?	No
19.	Audio/visual recording	Did the research use audio or visual recording to collect the data?	Data collection methods line 128-129 (Audio recorded)
20.	Field notes	Were field notes made during and/or after the interview or focus group?	Data collection methods line 129-130 (during interview)

No	Item	Guide questions/description	Reference
21.	Duration	What was the duration of the interviews or focus group?	Data collection methods line 121
22.	Data saturation	Was data saturation discussed?	Data collection methods line 124
23.	Transcripts returned	Were transcripts returned to participants for comment and/or correction?	No
**Domain 3: analysis and findings**			
Data analysis			
24.	Number of data coders	How many data coders coded the data?	Data analysis line 140-141 Two data coders
25.	Description of the coding tree	Did authors provide a description of the coding tree?	Data analysis section line 138-142
26.	Derivation of themes	Were themes identified in advance or derived from the data?	Data analysis section line 141<br>In advance and derived from the data
27.	Software	What software, if applicable, was used to manage the data?	Data analysis section line 138
28.	Participant checking	Did participants provide feedback on the findings?	Data analysis section line 146 (yes through stakeholder workshop)
Reporting			
29.	Quotations presented	Were participant quotations presented to illustrate the themes /	Result section<br>Line

No	Item	Guide questions/description	Reference
		findings? Was each quotation identified? e.*g. participant number*	169,176,194,225,236,254,264,274,284,301,310,317,333
30.	Data and findings consistent	Was there consistency between the data presented and the findings?	Yes
31.	Clarity of major themes	Were major themes clearly presented in the findings?	Yes Result section line 147 to 327
32.	Clarity of minor themes	Is there a description of diverse cases or discussion of minor themes?	Yes, data analysis section line 137 to 140
